# Terahertz tunable three-dimensional photonic jets

**DOI:** 10.1038/s41598-024-64158-6

**Published:** 2024-07-17

**Authors:** Behrooz Rezaei, Babak Yahyapour, Arash Darafsheh

**Affiliations:** 1 Department of Condensed Matter Physics, Faculty of Physics, University of Tabriz, Tabriz, Iran; 2 Department of Engineering Physics, Polytechnique Montreal, Montreal, QC Canada; 3 Department of Radiation Oncology, Washington University School of Medicine in St. Louis, St. Louis, MO 63110 USA

**Keywords:** Photonic jet, Bulk Dirac semimetal, Terahertz, 3D FDTD, Optics and photonics, Applied optics

## Abstract

Highly localized electromagnetic field distributions near the “shadow-side” surface of certain transparent mesoscale bodies illuminated by light waves are called photonic jets. We demonstrated formation of three-dimensional (3D) tunable photonic jets in terahertz regime (terajets, TJs) by dielectric micro-objects -including spheres, cylinders, and cubes-coated with a bulk Dirac semimetal (BDS) layer, under uniform beam illumination. The optical characteristics of the produced TJs can be modulated dynamically through tuning the BDS layer’s index of refraction via changing its Fermi energy. It is demonstrated that the Fermi energy of BDS layer has a significant impact on tuning the optical characteristics of the produced photonic jets for both TE and TM polarizations. A notable polarization dependency of the characteristics of the TJs was also observed. The impact of obliquity of the incident beam was studied as well and it was demonstrated that electromagnetic field distributions corresponding to asymmetric photonic jets can be formed in which the intensity at the focal region is preserved in a wide angular range which could find potential application in scanning devices. It was found that the maximum intensity of the TJ occurs at a non-trivial morphology-dependent source-angle.

## Introduction

Photonic nanojets (PNJs) were introduced as propagating narrow high-intensity electromagnetic beams generated at the “shadow-side” surface of dielectric micro-cylinders and -spheres with diameters larger than the incident plane-wave’s wavelength^1–4^. The PNJ concept was later generalized to micro-objects with other morphologies^5^, such as truncated spheres^6^, spheroids^7^, cuboids^8,9^, axicones^10^, gratings^11,12^, and non-symmetrical objects^13^, as well as other illuminating beams such as Gaussian^14^ and Bessel^15^ beams. PNJs have found potential applications in microscopy^16–19^, spectroscopy^20^, profilometry^21–27^, photolithography^28,29^, optical trapping^30^, optical waveguides^31–34^, and biophotonics^35–37^. Initially, PNJs were mainly investigated at the visible spectrum, but then they were extended to terahertz (THz) regime (named “terajets”, TJs)^38–43^ due to broad range of potential applications of the THz technology in medical and material sciences, as well as in communication, homeland security, and pharmaceutical industry^44–47^. Developing materials and structures for light manipulation in THz regime is an active area of research^48–51^.

Optical characteristics of PNJs and TJs are governed by the index of refraction, geometry, and size of the generating micro-object^52–55^ as well as the refractive index of the material around the micro-object^56^ and properties of the incident light^2,57^. Refractive index can be modulated through incorporation of core–shell geometries^58,59^, liquid crystals^60,61^, temperature-sensitive vanadium oxide (VO_2_) coating^62^, and graphene coating^63,64^ in dielectric micro-objects in order to produce tunable PNJs and TJs. Although these approaches allow dynamic control of the refractive index, they are associated with their own features and principles of operation, for example slow response time (typically on the order of milliseconds) in liquid crystals ^65^, control by precise change in the temperature in VO_2_ (a phase change material), and the need to fabricate graphene with single-atom-layer thickness. In this context, ability to engineer materials with tunable refractive index in order to dynamically realize tunable TJs is appealing.

Dirac semimetals have been the subject of lots of recent research due to their ability in controlling the light propagation^66–73^. Bulk Dirac semimetals (BDSs) are topological materials and considered as 3D analogs of graphene due to their linear dispersion relation. The dielectric function of a BDS can be controlled dynamically through altering its Fermi energy by introducing an electric potential difference^74^. For electrically gating and controlling the Fermi energy, the electrolyte gating by means of ion gel could be utilized. The ion gel as a novel material with high conductivity has been used to electrically control the Fermi energy in layers of BDS^75^ and chemical potential of graphene^76^. The properties of the ion gel makes it a suitable alternative for solid polymers and conventional media for electrical conduction applications such as gate material for use in field effect transistors. BDSs behave like a metal and a dielectric, when the frequency is below and above the Fermi energy, respectively. Their response time upon application of the external voltage is on the order of picoseconds (ps) to nanoseconds (ns)^77,78^.

Very recently, we studied tunability of the optical properties of two-dimensional (2D) TJs produced by a BDS-coated dielectric rod^79^. In this paper, through numerical simulation, we demonstrate the formation of tunable TJs in BDS-coated 3D dielectric objects, including spheres, cylinders, and cubes, by optimizing their geometrical parameters and BDS layer’s thickness. Optical properties of the generated TJs can be dynamically controlled via altering the Fermi energy of the BDS coating. The influence of the polarization of the incident light on the characteristics of the TJs is also studied. For an optimal value of the Fermi energy, the formation of TJs in those structures under an oblique incident beam is investigated. The obtained results demonstrate that the Fermi energy affects remarkably the optical properties of the TJs and by selecting an appropriate value of the Femi energy, the intensity of the TJ could be maintained over a wide angular range.

## Geometry of the simulation

**Figure 1:**
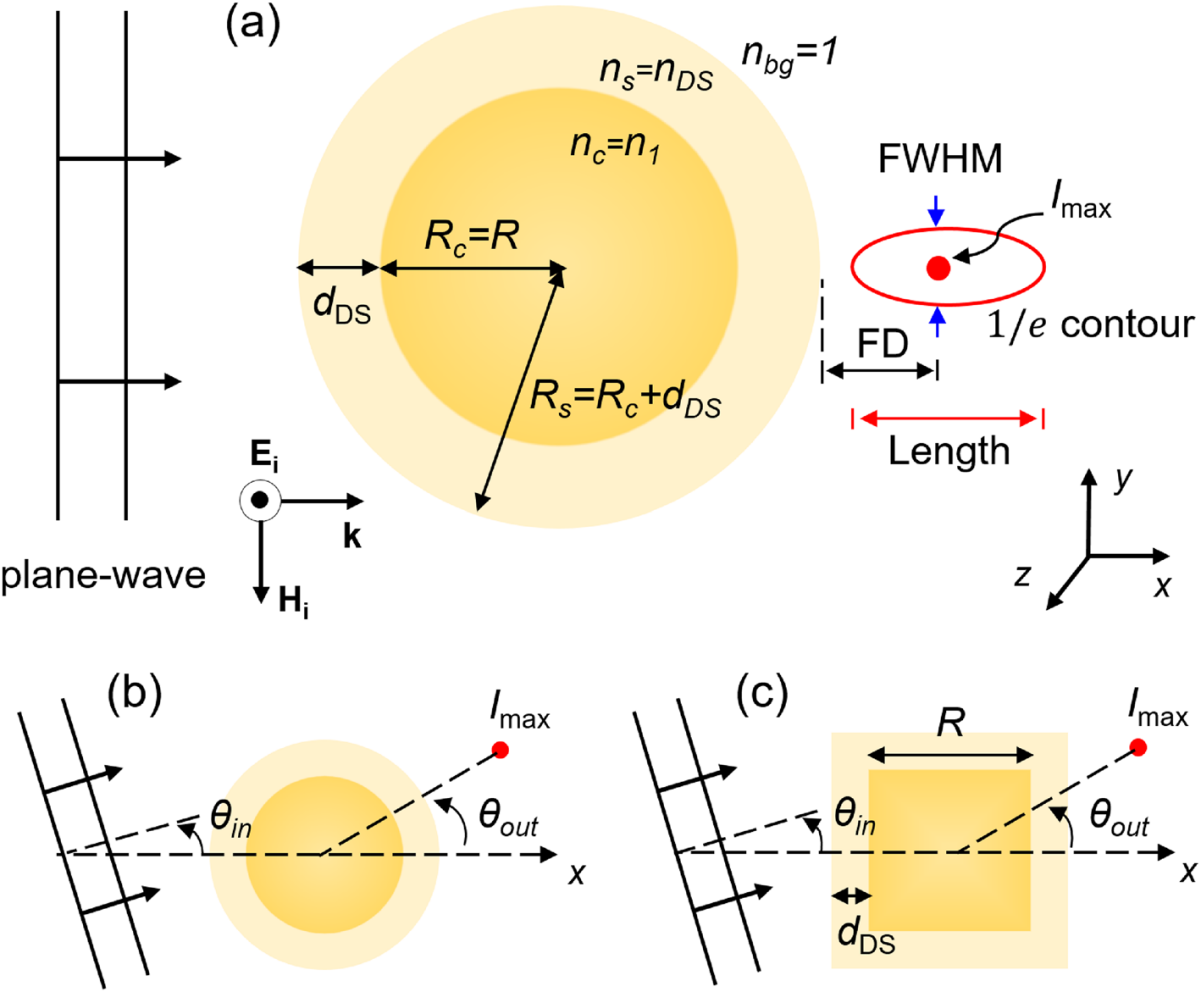
Schematic of the simulation geometry. A uniform beam, propagating along the *x*-axis, impinges upon a sphere (radius *R*), a cylinder (radius *R* and height* h*), and a cube (length R) with refractive index *n*_*1*_ coated with a BDS with thickness *d*_*DS*_ and index *n*_DS_. (**a**) Terajet characteristics include focal distance (calculated from the proximal surface of the particle to the locus of the maximum intensity), beam FWHM in *xy* plane, TJ length (measured as the distance between distal to proximal 1/$e$ intensity contour along the direction of the TJ, i.e. *x*-axis), and the maximum relative intensity. Geometry of oblique incident for a (**b**) sphere/cylinder and (**c**) cube. In the core–shell structure shown in panel (**a**), *n*_*c*_ (*R*_*c*_) and *n*_*s*_ (*R*_*s*_) represent the refractive indices (radii) of the core and shell, respectively. Drawings are not to scale.

We performed finite-difference time-domain (FDTD) simulations in 3D using Lumerical software package (Lumerical Inc., Canada). Figure 1 shows the schematic of the proposed structures, a sphere, cylinder, and cube made of high-density polyethylene (HDPE) with refractive index *n*_1_ = 1.51 at the THz frequency range^80^, surrounded by air (*n*_bg_ = 1) and covered with a uniform BDS layer of thickness *d*_*DS*_ and index *n*_*DS*_.

The quantitative key parameters of a typical PNJ or TJ are schematically presented in Fig. 1. Focal distance (FD) was defined as the distance from the proximal surface of the generating object to the focal point (locus of the maximum intensity) of the TJ. The TJ’s transverse size, quantified as the full-width at half-maximum (FWHM) in the *xy*-plane along *y*-axis, was calculated at the focal point. The TJ’s length was defined as the distance between proximal and distal 1/$e$ contour of the intensity along the beam direction (*x*-axis).

The dielectric function, $\varepsilon_{DS}$, of the BDS can be written as^67^:

1a $$\varepsilon_{DS} \left( {f,E_{f} } \right) = \varepsilon_{b} + i\frac{{\sigma_{DS} \left( {f,E_{f} } \right)}}{{2\pi f\varepsilon_{0} }} = \varepsilon_{b} - \frac{{{\text{Im}}\left\{ {\sigma_{DS} \left( {f,E_{F} } \right)} \right\}}}{{2\pi f\varepsilon_{0} }} + i\frac{{{\text{Re}}\left\{ {\sigma_{DS} \left( {f,E_{F} } \right)} \right\}}}{{2\pi f\varepsilon_{0} }}$$

in which $f$ is the frequency of the incident wave, $\varepsilon_{b}$ is the background’s effective dielectric constant, $\varepsilon_{0}$ is the permittivity of vacuum, $i = \sqrt { - 1}$, and $\sigma_{DS} \left( {f,E_{f} } \right)$ represents the BDS’s conductivity at a given frequency and Fermi Energy ($E_{f}$). The following equations give the complex optical conductivity of the BDS ^67^:

1b $${\text{Re}}\left\{ {\sigma_{DS} \left( {f,E_{F} } \right)} \right\}{ } = \frac{{e^{2} {\text{g}}k_{F} f}}{{12E_{F} }}\frac{{\sinh \left( {\frac{hf}{{2k_{B} T}}} \right)}}{{\cosh \left( {\frac{{E_{F} }}{{k_{B} T}}} \right) + \cosh \left( {\frac{hf}{{2k_{B} T}}} \right)}}$$

1c $${\text{Im}}\left\{ {\sigma_{DS} \left( {f,E_{F} } \right)} \right\} = \frac{{e^{2} {\text{g}}k_{F} }}{12\pi h}\left[ {\frac{{4E_{F} }}{hf}\left( {1 + \frac{{\pi^{2} }}{3}\left( {\frac{{k_{B} T}}{{E_{F} }}} \right)^{2} } \right) + \frac{8hf}{{E_{F} }}\mathop \smallint \limits_{0}^{{\varepsilon_{c} = 3}} \left( {\frac{{\frac{{\sinh \left( {\frac{{\varepsilon E_{f} }}{{k_{B} T}}} \right)}}{{\cosh \left( {\frac{{E_{F} }}{{k_{B} T}}} \right) + \cosh \left( {\frac{{\varepsilon E_{f} }}{{k_{B} T}}} \right)}} - \frac{{\sinh \left( {\frac{hf}{{2k_{B} T}}} \right)}}{{\cosh \left( {\frac{{E_{F} }}{{k_{B} T}}} \right) + \cosh \left( {\frac{hf}{{2k_{B} T}}} \right)}}}}{{\left( {\frac{hf}{{E_{f} }}} \right)^{2} - 4\varepsilon^{2} }}} \right)\varepsilon d\varepsilon } \right]$$

in which $k_{F} = E_{F} /\hbar v_{F}$ is the Fermi momentum, $v_{F} = 10^{6}$ m/s is the Fermi velocity, $\varepsilon$ = $E/E_{F}$, $\varepsilon_{c}$ = $E_{c} /E_{F}$, $E_{c}$ is the cut off energy above which the dispersion relation of the BDS becomes non-linear ($\varepsilon_{c}$ = 3 is our case), and $T = 300$ K is the temperature. Symbols $k_{B}$, *e,* $\hbar$, and *g* represent the Boltzmann constant, an electron’s charge, the reduced Planck constant, and the degeneracy factor (*g* = 4), respectively.

A uniform beam propagating along the *x*-axis, with unit amplitude, whose electric field oscillates along the i) *z*-axis (TE) and ii) *y*-axis (TM) was incident upon the proposed structures to investigate the dependency of the produced TJs on the polarization of the light source. The computational mesh size was Δ*x* = Δ*y* = Δ*z* = 0.3 μm (~ *λ*/140-*λ*/100) to secure converging results. The perfectly matched layers (PMLs) boundary condition (34-μm-thick) were employed around the simulation region to prevent spurious reflection ^81^. The incident uniform beam had a finite size encompassing the whole cross-sectional area of the object; its size along *y*-axis (*z*-axis) is 1200.3 μm (2201.4 μm) for sphere, 694.4 μm (680.9 μm) for cylinder, and 481.3 μm (691.8 μm) for the cube. The distance between the center of the source and center of the all dielectric sphere, cylinder, and the cube objects is 30 μm. Each run took about 8–9 h, 4–5 h, and 4–5 h for a sphere, cylinder, and cube, respectively, in our system with 64 GB RAM.

We numerically investigate the ability of the proposed BDS-coated dielectric objects (sphere, cubic and cylinder) to produce TJs, and control their optical properties by altering the BDS layer’s Fermi energy at room temperature. The BDS layer is considered to have similar properties to Cd_3_As_2_ (cadmium arsenide) and Na_3_Bi (trisodium bismuthide) with $\varepsilon_{b}$ = 12 and *g* = 4 ^67^. In contrast to 2D Dirac fermions in graphene or on the surface of 3D topological insulators, topological DSs possess 3D Dirac fermions in the bulk. Cd_3_As_2_ is a 3D DS material with separated Dirac points and linear energy dispersion in momentum space. It is a tetragonal material with space group I41/acd and lattice constants of $a = b = 1.26$ nm and $c \approx 2.54$ nm, which remains nearly cubic ($2a = 2b \approx c$) ^82^. This cell contains 96 cadmium atoms and 64 arsenic atoms with two cadmium vacancies. The Dirac equation describes the massless electrons in Cd_3_As_2_, suggesting a conical band that has twice degenerated due to spin. The crystal structure of Na_3_Bi, as a topological DS, is a body-centered tetragonal with space group of P4/mmm. The electronic band structure measurements and calculations of Na_3_Bi DS show a cone-shape with linear dispersion ^68^. The lattice constants of Na_3_Bi at the ground state are $a = 3.4116 A^{^\circ }$ and $c = 4.9530 A^{^\circ }$ ^83^.

Cd_3_As_2_, as a 3D BDS, has attracted research interests due to its chemical stability and extraordinary optical response^84^. It has been fabricated in thin films and at nanoscale through different techniques, including physical vapor deposition (PVD)^85^, pulse laser deposition (PLD)^86^, molecular beam epitaxy (MBE)^87^, chemical vapor deposition (CVD) ^88^, and self-selecting vapor growth (SSVG)^82^.

**Figure 2:**
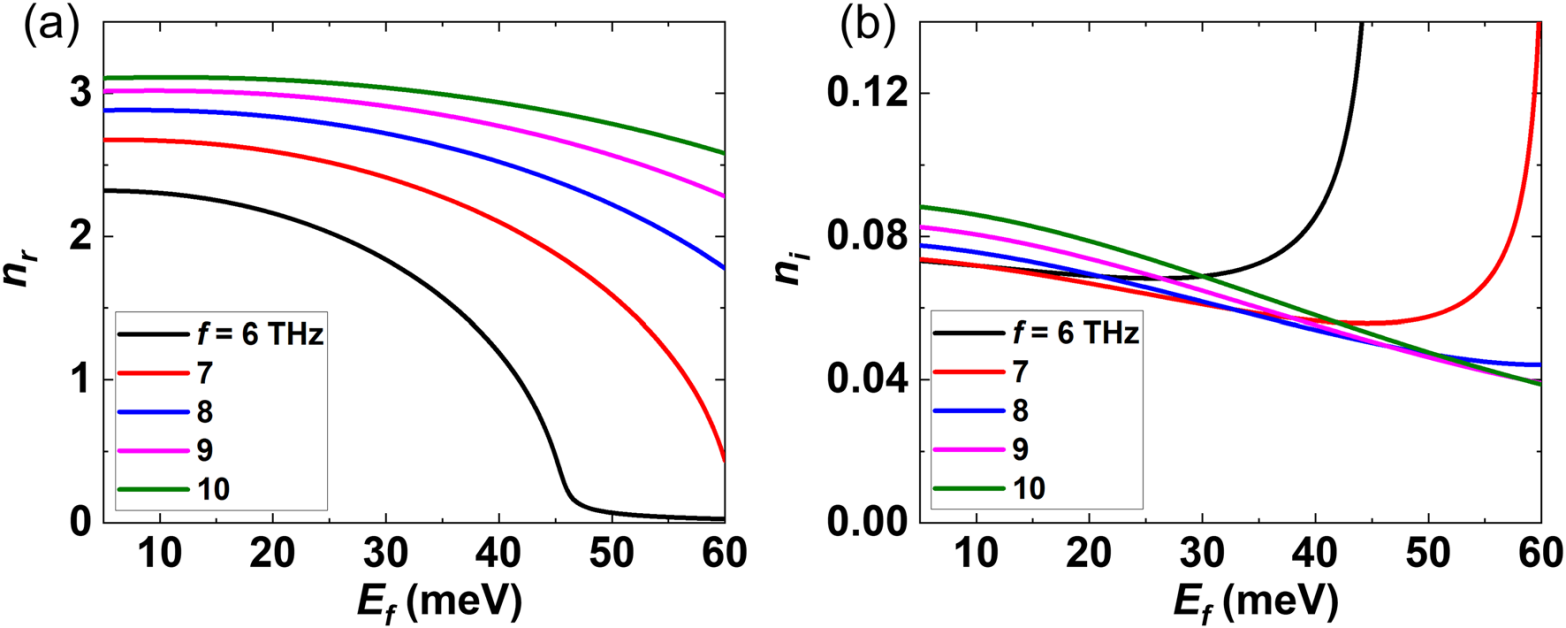
The index of refraction of a BDS, mimicking Na_3_Bi or Cd_3_As_2_, versus $E_{F}$ at several THz frequencies at room temperature: (**a**) real and (**b**) imaginary part.

The complex index of refraction of a non-magnetic medium is obtained through $\tilde{n}\left( \omega \right) = \sqrt {\varepsilon_{DS} \left( \omega \right)}$. The behavior of the real and imaginary index of refraction of the considered BDS versus the Fermi energy ($E_{F}$ = 5–60 meV) are plotted in Fig. 2 at different frequencies (6–10 THz). It can be seen that as the frequency decreases, the amount of change in the refractive index (dynamic range) over the range of Fermi energies increases, indicating higher degree of tunability of the produced TJs. However, at 6 THz, the real part of the index drops quickly to low values close to and smaller than unity. Hence, *f* = 7 THz (*λ* = 42.83 μm) which maintains a reasonably high index while providing the highest dynamic range was selected for all simulations in this work. The real part (*n*_*r*_) of the index decreases gradually with Fermi energy up to a certain energy beyond which it drops suddenly. The imaginary part (*n*_*i*_) of the refractive index decrease as the Fermi energy increases up to a certain energy beyond which it starts to increase. We chose $E_{F}$ = 10 meV as the lower end at which *n*_*r*_ = 2.669 and *n*_*i*_ = 0.071. At 7 THz and for $E_{F}$ > 55 meV, the real part of the index reaches unity, so $E_{F}$ = 55 meV was selected as the higher end at which *n*_*r*_ = 1.187 and *n*_*i*_ = 0.066. Due to a relatively low *n*_*i*_, the absorption associated with the BDS layer will be low in the selected Fermi energy range ($E_{F} \in$ ^10–55^).

It should be mentioned that in practice the $E_{F}$ and consequently the BDS’s index can be modulated dynamically through applying a voltage^89–92^, or by an alkaline surface doping ^68,93^. In this way, the propagation of light can be modulated by the BDS-coated objects, affecting the generated TJs’ characteristics.

In order to demonstrate the formation of tunable 3D TJs, we considered three cases: (i) a BDS-coated dielectric sphere with radius *R* = 500 μm (~ 11.7λ) and thickness of the BDS layer *d*_*DS*_ = 1.5 μm (~ 0.035λ); (ii) a cylinder with radius *R* = 300 μm (~ 7λ), height *h* = 180 μm (~ 4.2λ), and thickness of the BDS layer *d*_*DS*_ = 1.5 μm; (iii) a cube with side length *R* = 300 μm and thickness of the BDS layer *d*_*DS*_ = 1.5 μm. The thickness of the BDS layer was kept small in all cases to minimize the absorption. The object’s size for each case was obtained through simulating objects with sizes in the range 2*λ* up to 25*λ*, where *λ* is the wavelength, with 0.1*λ* increments and selecting the size that produced the highest intensity TJ. This is the size range over which production of photonic jets is more efficient ^53^. Next, the BDS layer with different thicknesses between 0.5–2 μm, with 0.1 μm increments, was added and the optimum thickness was selected. Here, there is a trade-off between refractive power of the layer and its absorption imposed by the imaginary component of the refractive index.

Next, we studied the impact of the obliquity of the incident uniform beam on the TJ formation in the abovementioned cases. Figure 1b,c show the geometry of the oblique incident case on a sphere/cylinder and a cube, respectively. The input source angle was defined as the rotation angle of the source along *x*-axis as represented by *θ*_*in*_ in Fig. 1b,c. The emergence angle of the TJ was defined as the angle between the *x*-axis and the line passing through the location of the maximum intensity outside of the object and the center of the object as shown by *θ*_*out*_ in Fig. 1b,c. Other parameters of the simulation remained unchanged.

## Results and discussions

**Figure 3:**
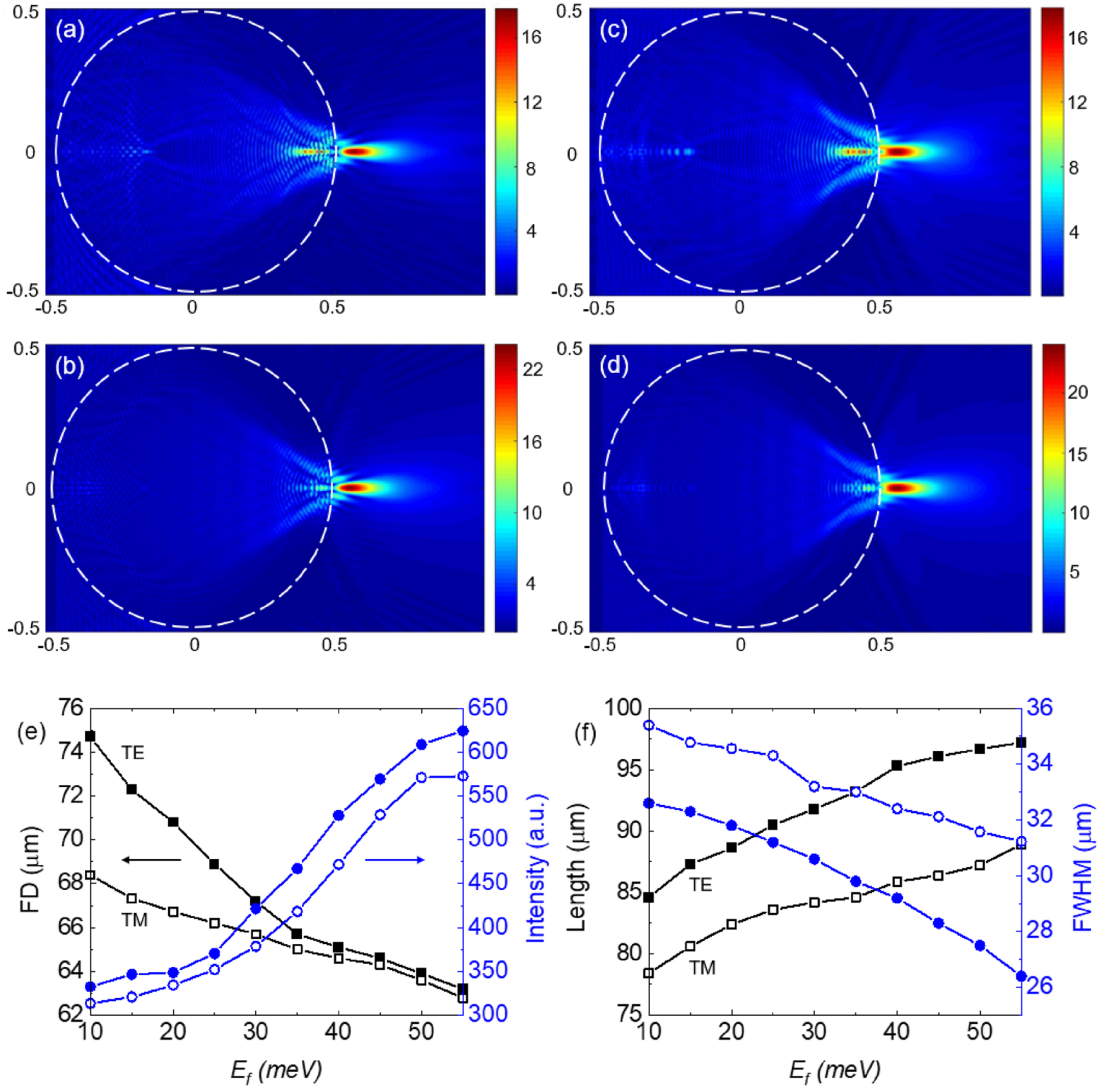
The electric field distribution inside and around a BDS-coated dielectric sphere with *R* = 500 μm and *d*_*DS*_ = 1.5 μm at *T* = 300 K for (**a**) $E_{F}$ = 10 meV (TE), (**b**) $E_{F}$ = 55 meV (TE), (**c**) $E_{F}$ = 10 meV (TM), and (**d**) $E_{F}$ = 55 meV (TM). (**e**) TJ’s calculated FD (square symbols) and intensity (circle symbols) versus $E_{F}$. (**f**) TJ’s length (square symbol) and FWHM (circle symbol) versus $E_{F}$. Hollow (solid) symbols correspond to TM (TE) polarization of the incident uniform beam.

The distribution of the electric field of the TJs produced by the BDS-coated sphere at Fermi energies of $E_{F}$ = 10 meV and $E_{F}$ = 55 meV, for TE-polarized incident wave, are presented in Fig. 3a,b, respectively. The corresponding results for the TM-polarized case are presented in Fig. 3c,d, respectively. The modulation of the characteristics of the produced TJs, realized through changing the BDS layer’s Fermi energy, are presented in Fig. 3e,f. Since the intensity is proportional to the second power of the electric field distribution, in order to calculate the TJ’s FWHM, the location of the maximum intensity is identified first. Next the intensity profile along a line parallel to the *y*-axis passing through the location of the maximum intensity is obtained. We observed a decreasing (increasing) trend in FD and length (intensity and FWHM) of the TJs as the Fermi energy increases. For example, for the TM (TE) incident wave, the FD decreased from 68.4 (74.7) μm to 62.8 (63.2) μm when the $E_{F}$ of the BDS layer was increased from 10 to 55 meV, providing 5.6 (11.5) μm dynamic range. Also, within the same Fermi energy range, the enhancement in intensity of the TJ was increased from 313.5 (332.5) to 572.9 (624.4) for the TM (TE) case. The FWHM decreased from 35.4 (32.6) μm to 31.23 (26.4) μm while the TJ length increased from 78.4 (84.6) μm to 88.9 (97.2) μm in which the corresponding amounts of tuning were 4.17 (6.2) μm and 10.5 (12.6) μm, respectively. It can be seen that the TE case provides more dynamic range for the tuning compared to the TM case. The focal distance and TJs are longer, more intense, with smaller FWHM in TE cases compared to the TM cases. It has been suggested that TM polarization generates wider PNJs compared to the TE polarization due to longitudinal component of the TM-polarized incident light^94^.

**Figure 4:**
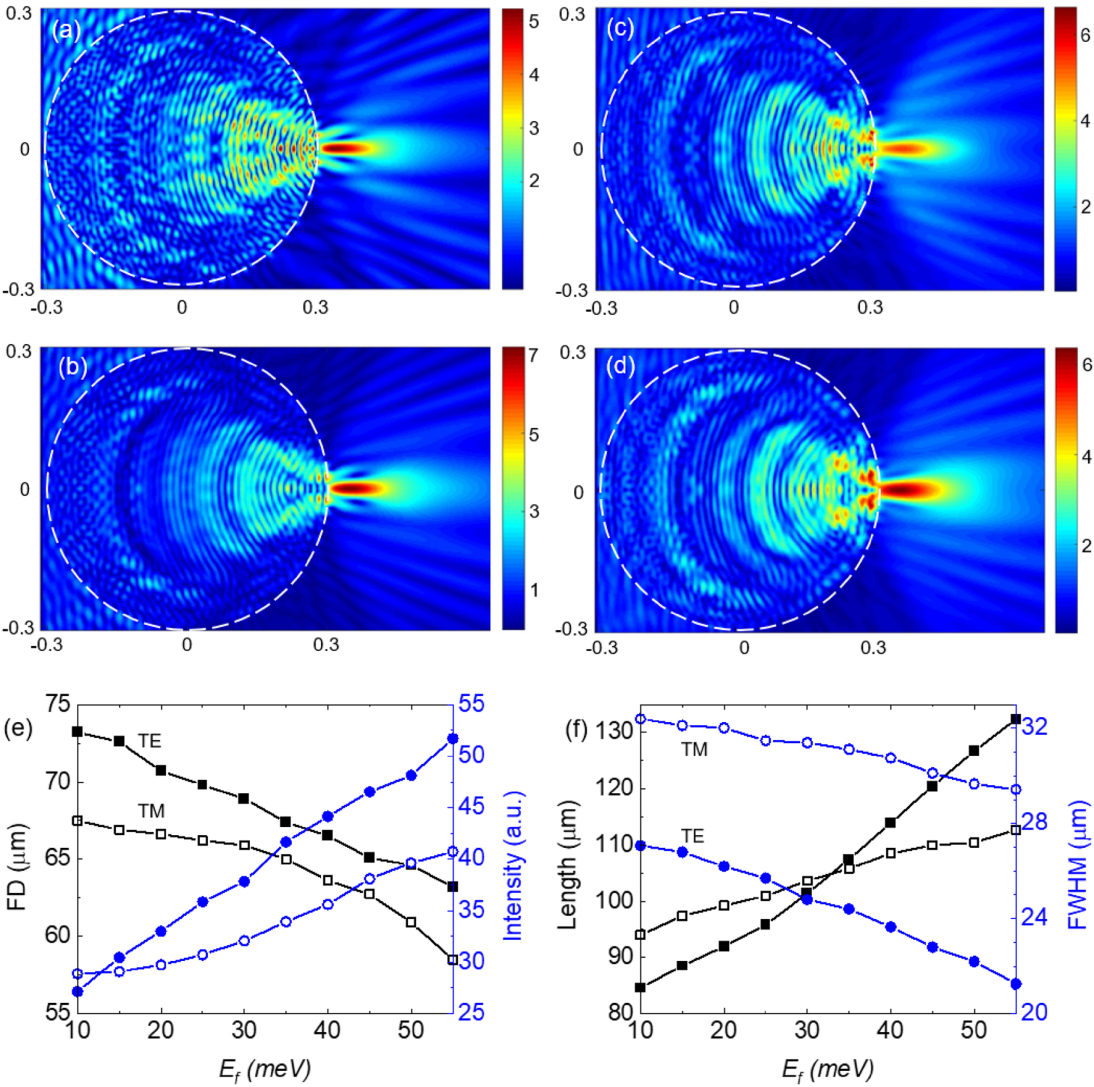
The distribution of the electric field inside and around a BDS-coated cylinder with *R* = 300 μm and *d*_*DS*_ = 1.5 μm at *T* = 300 K for (**a**) $E_{F}$ = 10 meV (TE), (**b**) $E_{F}$ = 55 meV (TE), (**c**) $E_{F}$ = 10 meV (TM), and (**d**) $E_{F}$ = 55 meV (TM). (**e**) TJ’s FD (square symbols) and intensity (circle symbols) versus $E_{F}$. (**f**) TJ’s calculated length (square symbols) and FWHM (circle symbols) versus $E_{F}$. Hollow (solid) symbols correspond to TM (TE) polarization of the incident uniform beam.

The distribution of the electric field of the TJs generated by the cylinder at $E_{F}$ = 10 meV and $E_{F}$ = 55 meV, for TE polarized incident wave, are shown in Fig. 4a,b, respectively. The corresponding cases for the TM-polarized case are presented in Fig. 4c,d, respectively. The plots of the TJ’s FD and intensity, as a function of $E_{F}$, are presented in Fig. 4). The plots of the TJ’s FWHM and length, as a function of $E_{F}$, are presented in Fig. 4f. It can be seen that the optical characteristics of the produced TJs follow a similar trend as in the corresponding sphere cases in terms of the Fermi energy. However, the sphere provided TJs with an order of magnitude more intensity. The intensity enhancement of the TJ varied between 28.9 (27.2) and 40.75 (51.7) for TM (TE) case. The amount of tuning for FD is 9 μm (10 μm), which varies from 67.5 μm (73.2 μm) to 58.5 μm (63.2 μm) for the TM (TE) case. The FWHM varied between 29.4 μm (21.3 μm) and 32.4 μm (27.1 μm) for TM (TE) case. The TJ length varied between 94 μm (84.6 μm) and 112.7 μm (132.4 μm). The TE case provided more dynamic range for the tunability compared to the TM case.

Using geometrical optics paraxial approximation ^18^, we can calculate the focal distance of a core–shell system with circular cross-section with the core and shell radii of $R_{c}$ and $R_{s}$ and refractive indices of $n_{c}$ and $n_{s} ,{\text{ respectively}}$, as:

2 $$FD = \frac{{R_{s} \left( {2R_{s} n_{c} - 2R_{c} n_{c} - 2R_{s} n_{s} + R_{c} n_{c} n_{s} } \right)}}{{2\left( { - R_{s} n_{c} + R_{c} n_{c} + R_{s} n_{s} - R_{c} n_{c} n_{s} } \right)}}$$

For our core–shell spheres with $R_{c}$ = 500 μm and $R_{s}$ = 501.5 μm, we have $FD$ = 242.8 μm and 239.7 μm at $E_{F}$ = 10 meV and $E_{F}$ = 55 meV, respectively. For the same sphere without the shell, the *FD* is 240.2 μm. In our core–shell structures composed of concentric spheres and co-axial cylinders, the core due to its significantly larger size influences the focusing more than the shell does. Obviously, we do not expect to accurately calculate the characteristics of mesoscale objects using geometrical optics. However, it is interesting to note that the trend is in agreement with the FDTD simulation results. As the shell index decreases with the increase of the *E*_*f*_, the *FD* decreases. TJ formation is a complex interplay of the incoming, refracted, and diffracted waves inside and around the micro-object. Generally, smaller spheres and cylinders produce PJs closer to the distal interface, i.e. shorter FD, with smaller FWHM; also, higher refractive index of the core, would lead to shorter FD and smaller FWHM of the produced PJ ^2^. In principle, these parameters can be further exploited to optimize the TJs.

In our previous work for 2D cases, we observed intensity enhancement factors in the range of ~ 9.5–22, here for the 3D cylinders the intensity enhancement factor is ~ 41–52. In addition to the refracted waves and diffracted waves at the periphery of the cylinder, the diffracted waves from the sides also contribute to the focal point’s intensity in the 3D case.

**Figure 5:**
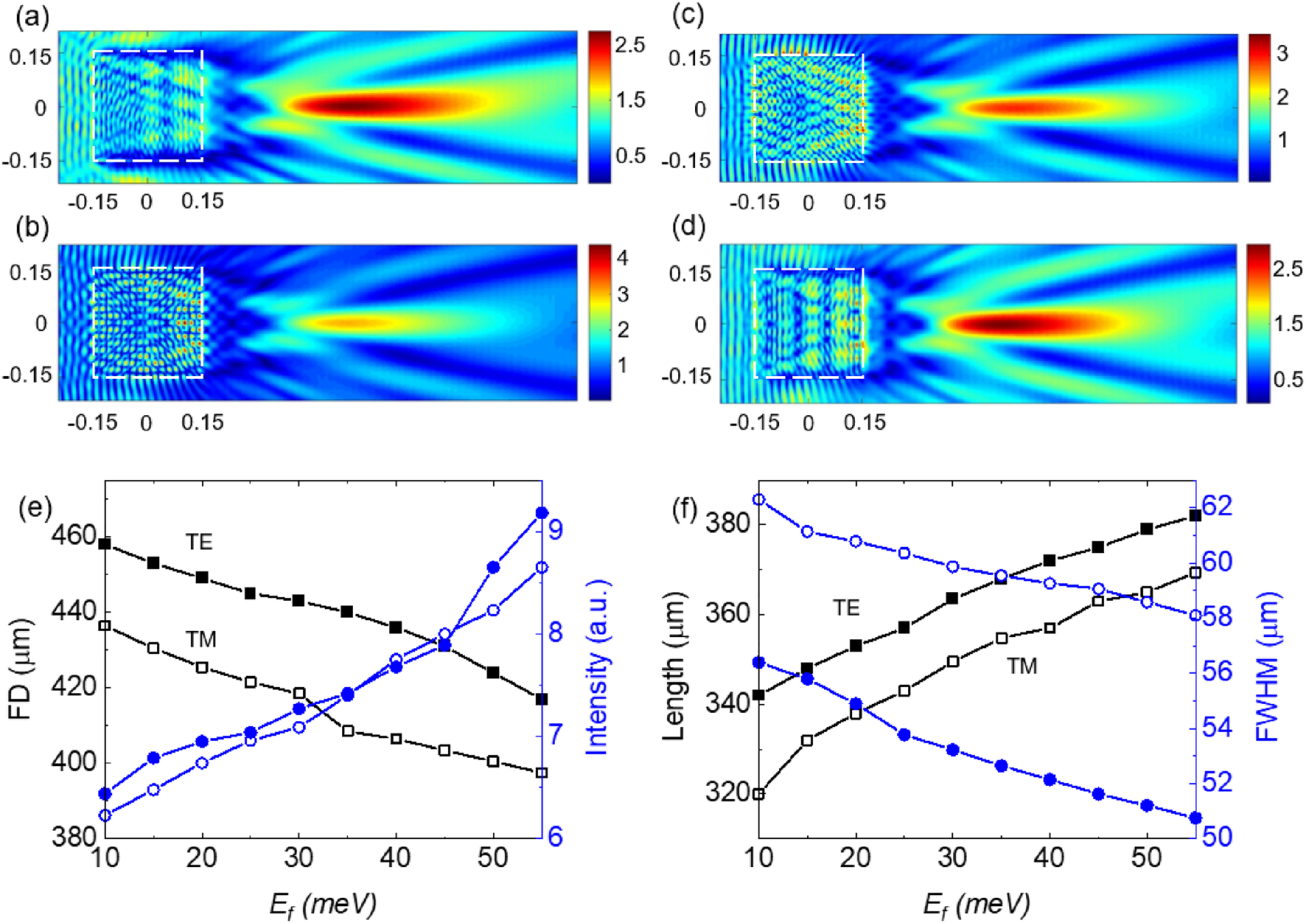
Distribution of the electric field distribution around and inside a BDS-coated cube with *R* = 300 μm and *d*_*DS*_ = 1.5 μm at *T* = 300 K for (**a**) $E_{F}$ = 10 meV (TE), (**b**) $E_{F}$ = 55 meV (TE), (**c**) $E_{F}$ = 10 meV (TM), and (**d**) $E_{F}$ = 55 meV (TM). (**e**) Plot of FD (square symbols) and intensity (circle symbols) vs. $E_{F}$. (**f**) TJ’s length (square) and FWHM (circle) versuss $E_{F}$. Hollow (solid) symbols correspond to TM (TE) polarization of the light source.

Figure 5 shows how the optical properties of a TJ produced by a cube changes as the $E_{F}$ of the BDS coating changes. The distribution of the electric field of the TJs, for the TE-polarized incident wave, at $E_{F}$ = 10 meV and $E_{F}$ = 55 meV are shown in Fig. 5a,b, respectively. The quantitative properties of the TJs for the TM-polarized light source as a function of the $E_{F}$ are presented in Fig. 5c,d showing a similar trend as in the sphere and cylinder cases. However, the intensity in the cube-generated TJs is less than those produced by cylinders/spheres and the focal distance is longer due to the weaker focusing effect in the cube. The intensity enhancement of the TJ varied between 6.2 (6.4) and 8.6 (9.2) for TM (TE) case. The FD varies between 436.5 (458) μm and 397.5 (417) μm corresponding to 39 (41) μm tuning for TM (TE) case. Within the same Fermi energy, the FWHM with a tuning of 4.2 μm (5.6) reduces from 62.3 (56.4) μm to 58.1 (50.8) μm, while the length of TJ with a tuning of 49.4 (40) μm increased from 320 (342) μm to 369.4 (382) μm for TM (TE) case. The TE case provided more dynamic range for the tunability compared to the TM case for the intensity, focal distance, and the spot size. The TJ length showed more dynamic range for the TM case compared to the TE case.

In most of the cases, the focal distance and TJs are longer, more intense with smaller FWHM in TE cases compared to the TM cases. When comparing the findings for all 3D TJs, the highest tuning of the intensity is attained for spheres, while the tuning of the TJ's length is more noticeable for the cube. In all cases, as the *E*_*f*_ increases, the intensity of the TJ increases. It can be in part explained by the fact that since the index of the BDS coating decreases as *E*_*f*_ increases, the Fresnel losses at each interface decrease which increases the photon fluence at the focal point. The intensity of the TJ is highest for the sphere due to its extended curved boundary compared to the cylinder and cube that provides high photon fluence at the focal point. The TJ lengths are longer in the cubes (cylinders) than those in the cylinders (spheres). A quick convergence of the Poynting vector around the focal spot would produce more intense TJs with shorter length ^95^. However, when the Poynting vector is less diverging and remains parallel near the focal spot, the TJ is more elongated with longer FD as in the cube case.

**Figure 6:**
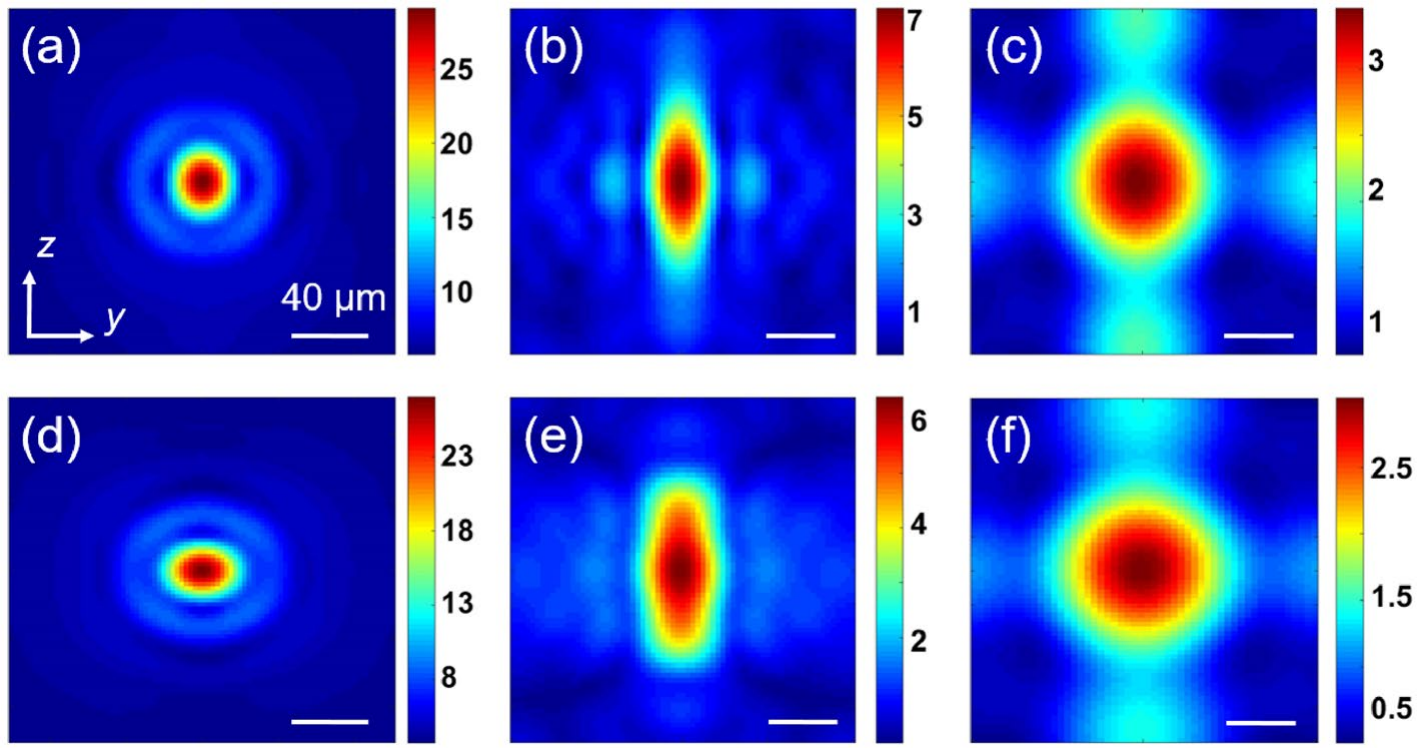
Distribution of the electric field inside and around a BDS-coated (**a**, **d**) sphere (*R* = 500 μm and *d*_*DS*_ = 1.5 μm), (**b**, **e**) cylinder (*R* = 300 μm and *d*_*DS*_ = 1.5 μm), and (**c**, **f**) cube (*R* = 300 μm and *d*_*DS*_ = 1.5 μm) at *T* = 300 K and $E_{F}$ = 55 meV. Polarization of the incident wave is along the (**a**–**c**) *z*-axis (TE) and (**d**–**f**) *y*-axis (TM). Scale bars are 40 μm.

Figure 6 shows the TJ’s electric field distribution (for $E_{F}$ = 55 meV) in the beam’s eye-view, i.e. *yz*-plane encompassing the locus of maximum intensity. The TJ’s FWHM along the *y*-axis is what we have reported so far. The TJ’s FWHM was calculated along the *z*-axis as well for these cases presented in Fig. 6. The FWHM values along *y*-axis (*z*-axis) are 31.2 (25.7), 29.4 (69.7), and 58.1 (62.4) μm for the sphere, cylinder, and cube, respectively, under normal incidence and TM polarization of the uniform beam. The corresponding values for the TE case are 26.4 (32.8), 21.3 (49.3), and 50.8 (66) μm. It can be seen that the TJ distribution is not totally symmetric, a consequence of vectorial nature of the incident uniform beam. According to Richards and Wolf diffraction theory ^96^, under high numerical aperture focus, the intensity profile of the focal spot would be elongated along the direction of the polarization. Here we see that effect in the produced TJs; for TE (TM) case elongation of the TJ can be seen along *z*-axis (*y*-axis).

**Figure 7:**
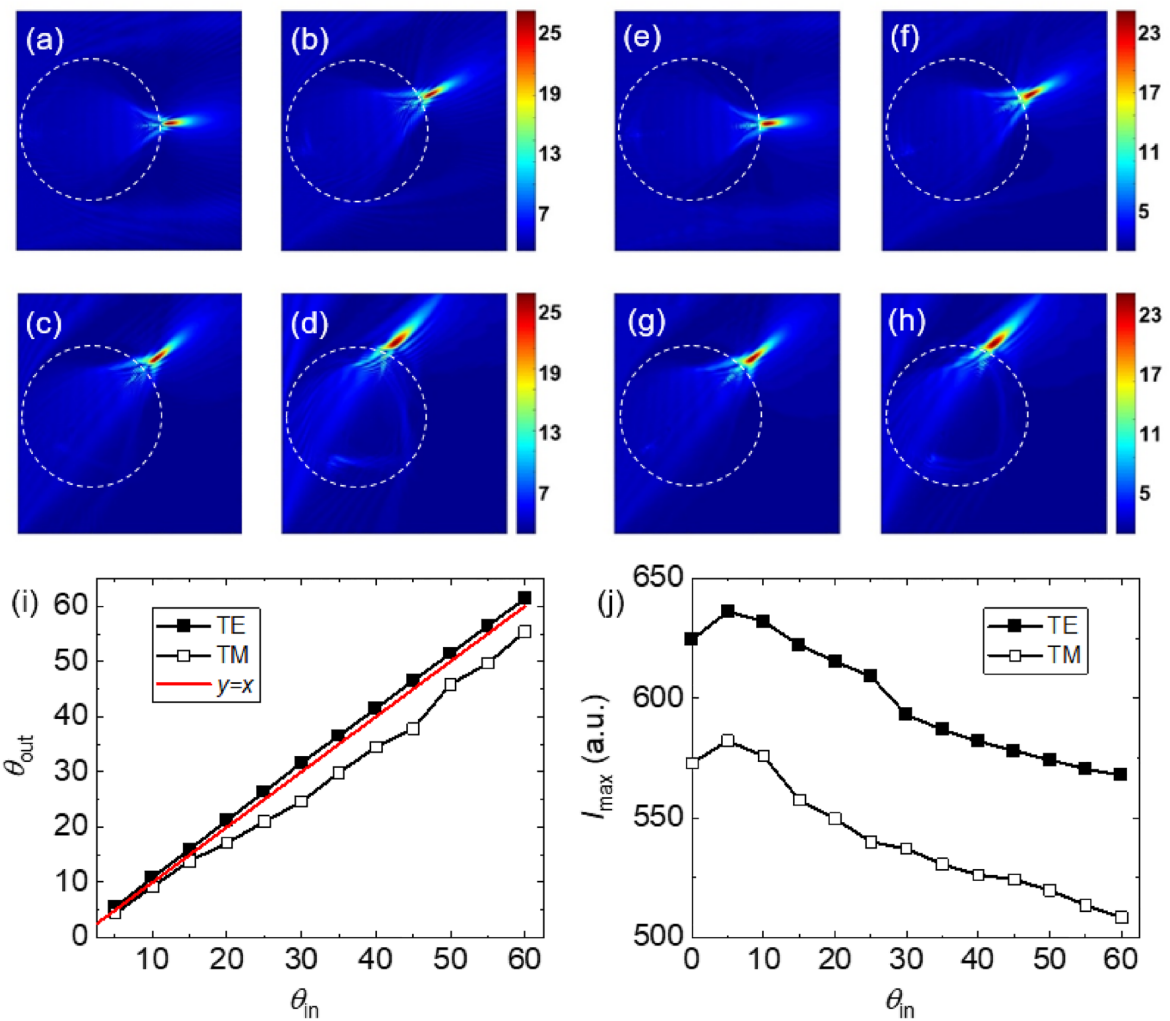
(**a**–**d**) The electric field distributions of the TJ formed by a BDS-coated dielectric sphere with *R* = 500 μm and *d*_*DS*_ = 1.5 μm at Fermi energy of $E_{F}$ = 55 meV for input angles of (**a**) 5°, (**b**) 25°, (**c**) 40° and (**d**) 60° for the TE case. (**e**–**h**) The TM counterparts of (**a**–**d**). The dashed circles show the boundary of the sphere. (**i**, **j**) The emergence angle and intensity of the TJ, respectively, as a function of the uniform beam rotation angle.

Next, we analyzed the optical properties of BDS-coated dielectric objects under oblique incidence to verify the robustness of the produced TJs at various angles of the incident beam. We found that the intensity of the TJ was maximum at $E_{F}$ = 55 meV within the range of studied Fermi energies. Figure 7a–d illustrate the simulation results of electric field distribution in the *xy*-plane for source rotation angles of 5°, 25°, 40°, and 60° for the TE case. The corresponding results for the TM case are presented in Fig. 7e–h. When the source angle was altered, the TJ was deflected, and the intensity at the focused region was maintained over a large angular range, demonstrating the micro-object’s ability to work as a wide-scanning TJ-focusing lens. When the angle of the incident uniform beam was varied, the numerical results for deflection angle of the TJ produced by the sphere is presented in Fig. 7i. The intensity as a function of the rotation angle of the incident uniform beam is shown in Fig. 7j. It should be noted that the uniform beam source was rotated along the *x*-axis which caused the asymmetric appearance of the TJs. It should be noted that the asymmetry is caused by the finite size of the incident uniform beam that leads to partial illumination of the object. Interestingly, it can be seen that the maximum intensity of the TJ occurs at ~ 5° source rotation angle, not necessarily at normal incidence. Introduction of an asymmetric optical path length along the optical axis due to the obliquity of the incident beam produces an asymmetric TJ as a result of difference between phase velocity and the interference of the waves inside the particle.

**Figure 8:**
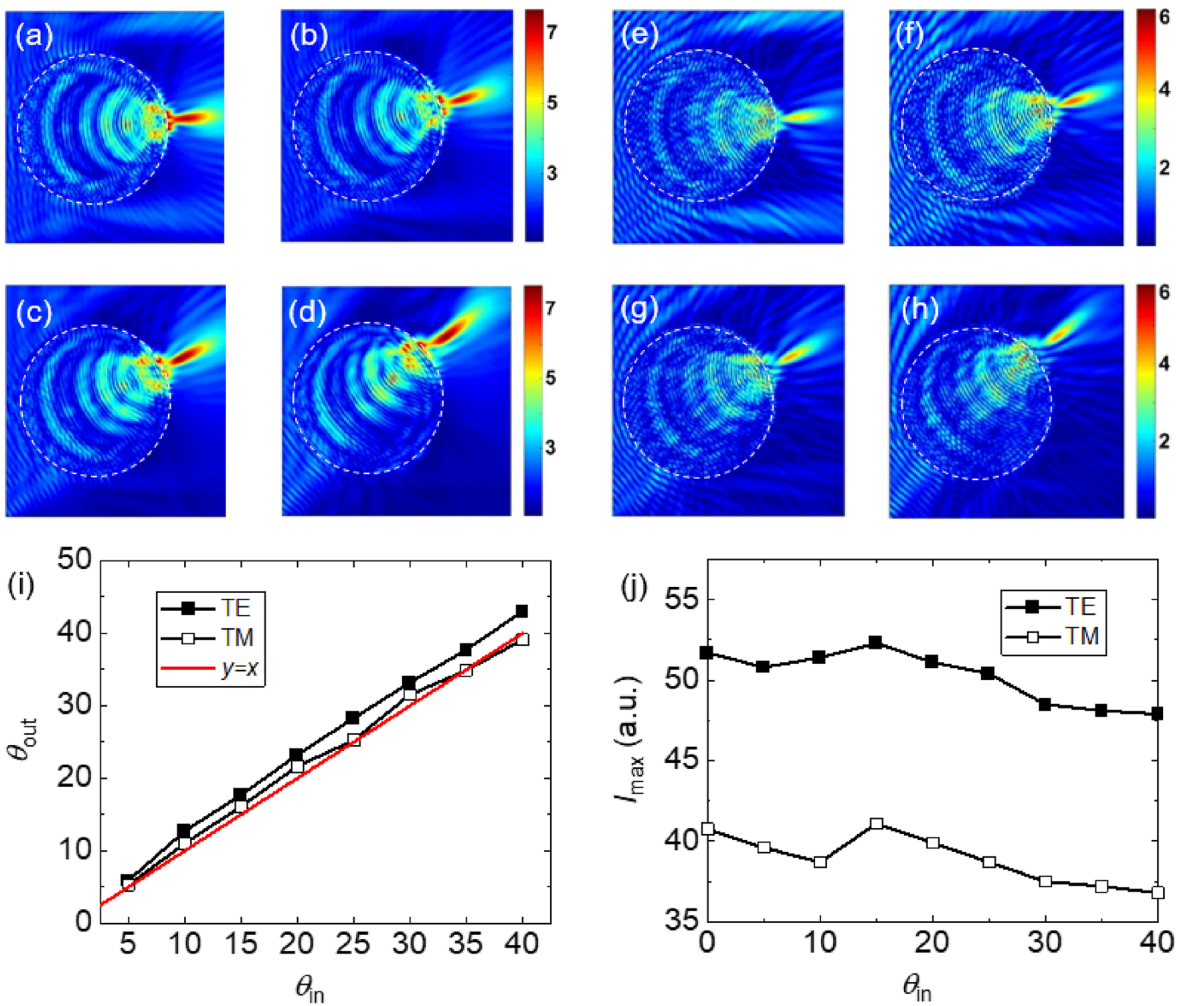
(**a**–**d**) The electric field distributions of the TJ formed by a BDS-coated dielectric cylinder at Fermi energy of $E_{F}$ = 55 meV for source rotation angles of (**b**) 5°, (**c**) 15°, (**d**) 25°, and (**e**) 40° for the TE case. (**e**–**h**) The TM counterparts of (**a**–**d**). The dashed circles represent the boundary of the cylinder. (**i**, **j**) The emergence angle and intensity of the TJ, respectively, as a function of the uniform beam rotation angle.

The simulation results for the cylinder case at $E_{F}$ = 55 meV are presented in Fig. 8 indicating how the TJ is deflected by changing the source rotation angle. Figure 8a–d represent the electric field distribution in *xy*-plane for input angles of 5°, 15°, 25°, and 40° for TE case. The corresponding results for the TM case are presented in Fig. 8e–h. Figure 8i shows the dependency of the deflected angle of the TJ versus the source rotation angle. The intensity as a function of the source rotation angle is shown Fig. 8j. The observed asymmetry in the TJ shape is due to the fact that the source was rotated along the *x*-axis. Similar to the sphere case, it can be seen that the maximum intensity of the TJ occurs at a non-trivial source rotation angle, here at ~ 15° source rotation angle.

**Figure 9:**
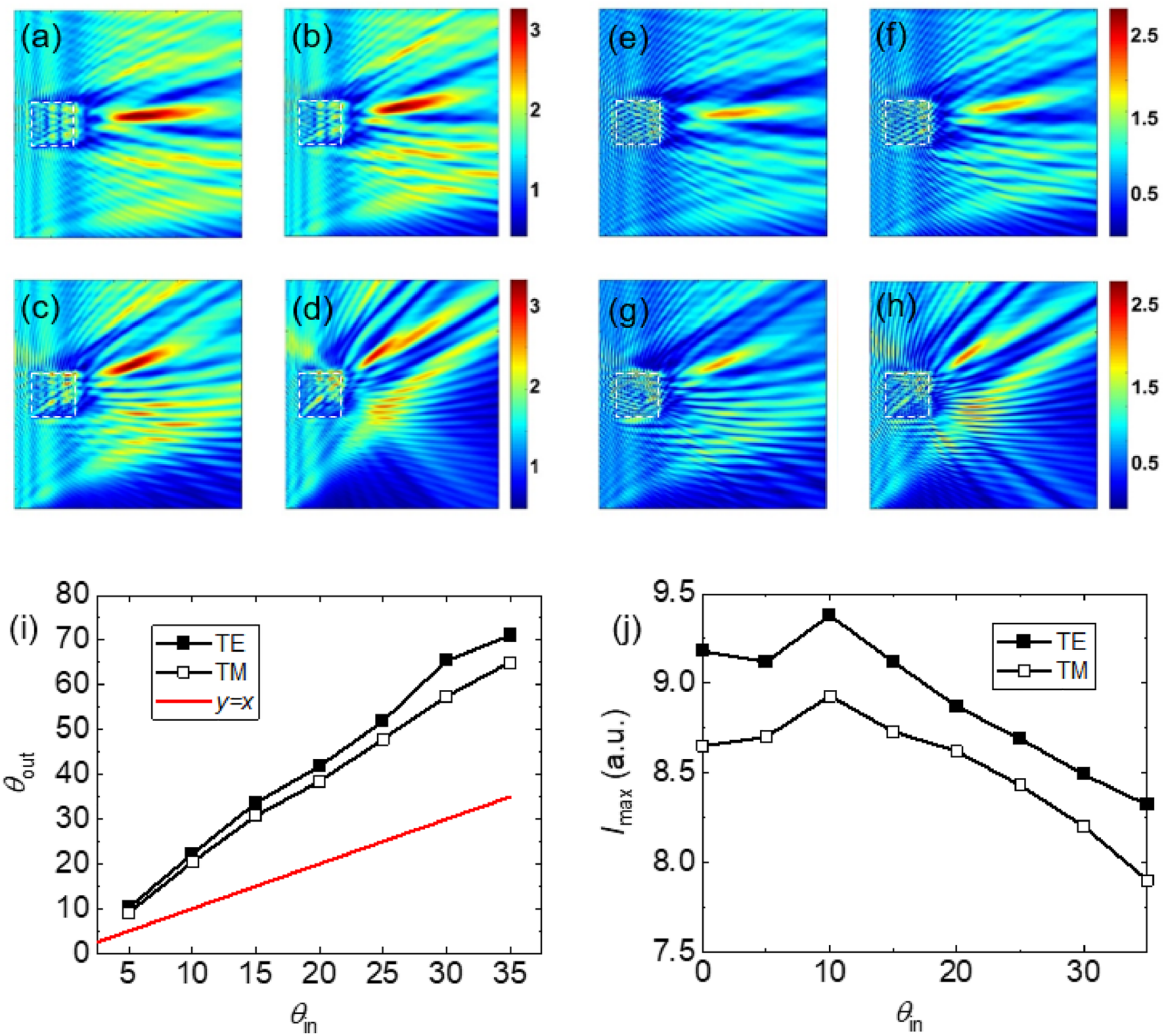
(**a**–**d**) The electric field distributions of the TJ formed by a BDS-coated dielectric cube with *R* = 300 μm and *d*_*DS*_ = 1.5 μm at Fermi energy of $E_{F}$ = 55 meV for source rotation angles of (**a**) 5°, (**b**) 10°, (**c**) 20°, and (**d**) 35° for the TE case. (**e**–**h**) The TM counterparts of (a-d). The dashed squares represent the boundary of the cube. (**i**, **j**) The output angle and intensity of the TJ, respectively, as a function of the uniform beam rotation angle.

The simulation results for the 3D BDS-coated cube at $E_{F}$ = 55 meV is shown in Fig. 9. Figure 9a–d show the electric field distribution for input angles of 5°, 10°, 20°, and 35° for the TE case. The corresponding results for the TM case are presented in Fig. 9e–h. Figure 9i,j show the relationship between the output angle and intensity as a function of the input angle. Similar to the sphere and cylinder cases, it can be seen that the maximum intensity of the TJ occurs at a morphology-dependent source rotation angle, here at ~ 10°.

It can be seen in Figs. 7, 8, 9 that the exit angle of the TJs in the sphere and cylinder cases follow a somewhat symmetric trend with respect to the incident angle due to the inherent circular symmetry of the producing micro-object. However, the asymmetry between *θ*_*in*_ and *θ*_*out*_ in the cube case is mostly stems from the lack of rotational symmetry of the cube compared to the sphere/cylinder.

A list of PJ characteristics produced using different objects at different wavelengths is provided in Table 1. These parameters should scale with the wavelength following Maxwell’s equations. A distinct feature of the current work with the majority of the previous works is that the PJs are dynamically tunable. Depending on the application, operation wavelength, and choice of available materials, an ad hoc simulation must be performed to select the optimum PJ-producing geometry.

**Table 1:** Characteristics of photonic jets produced by objects with various shapes and sizes.

Shape	Wavelength (λ) (µm)	Size (D/λ)	Index contrast	FWHM/λ	Intensity enhancement factor (I_max_/I_0_)	Jet length/λ	Tunable	References
DS-coated 3D sphere	42.83	23.34	*n*_c_ = 1.51	0.76–0.62 (TE) 0.83–0.73 (TM)	332.5–624.4 (TE) 313.5–572.9 (TM)	1.97–2.27 (TE) 1.83–2.07 (TM)	Yes	This work
DS-coated 3D cylinder	42.83	~ 4.2 to 14	*n*_c_ = 1.51	0.63–0.50 (TE) 0.76–0.69 (TM)	27.2–51.7 (TE) 28.9–40.75 (TM)	1.97–3.09 (TE) 2.19–2.63 (TM)	Yes	This work
DS-coated 3D cube	42.83	7	*n*_c_ = 1.51	1.32–1.19 (TE) 1.45–1.36 (TM)	6.4–9.2 (TE) 6.2–8.6 (TM)	7.98–9.92 (TE) 7.47–8.62 (TM)	Yes	This work
DS-coated 2D cylinder	37.47	19.2	*n*_c_ = 1.44	~ 1.03 to 0.56	8.8–17.5	~ 9.3 to 6.2	Yes	^79^
DS-coated 2D cylinder	37.47	30.4	*n*_c_ = 1.44	~ 0.93 to 0.67	9.45–22.4	~ 5.84 to 3.82	Yes	^79^
DS-coated 2D cylinder	33.33	21.6	*n*_c_ = 1.44	~ 0.97 to 0.7	11.1–20.1	~ 5.3 to 3	Yes	^79^
DS-coated 2D cylinder	42.83	23.4	*n*_c_ = 1.44	~ 0.7 to 0.65	15.9–22.4	~ 7.5 to 8.4	Yes	^79^
Circular LC core–shell	0.676	5	1.738	~ 0.45 to 0.34	~ 0.7 to 1	~ 3.6 to 1.8	Yes	^61^
Elliptical LC core–shell	0.676	5	1.738	~ 0.7–0.32	~ 0.3 to 1	~ 10.4 to 1	Yes	^61^
VO_2_-coated sphere	0.8	~ 6.34	1.5	0.5375–0.4625	22.7	N/R	Yes	^62^
Cylinder	0.2–0.5	10–33.33	1.7–1.75	0.5–0.66	N/R	1.6–3.33	No	^1^
Sphere	0.4	2.5–20	1.59	0.325–525	N/R	N/R	No	^54^
Sphere	0.9	~ 3.5 to 4.4	1.49	0.41–0.78	~ 8–17	2.3–9.3	No	^14^
Sphere	0.532	~ 10.8	1.5	~ 0.5	~ 200	N/R	No	^97^
Axicon	1	~ 6 to 7	1.5	0.3	~ 50	N/R	No	^98^
Axicon	0.532	1–6	1.46	~ 0.45 to 0.79	~ 20 to 40	~ 3.5 to 5.5	No	^13^
Axicon	0.532	~ 1.9 to 5.6	1.5	~ 0.45 to 0.79	Up to 40	Up to 5.5	No	^10^
Cube	3000	1.2	1.75	< 0.4	~ 15	N/R	No	^9^
Cube	3000	1.2	1.41	0.46	~ 10	N/R	No	^9^
Cuboid	0.532	0.5–6	1.46	~ 0.43 to 1.75	N/R	Up to 25	No	^8^
Cuboid (reflection)	Scaled	0.2–1	1.46	~ 0.4	Up to 17	1.08	No	^39^
Cuboid array	8570	1	1.46	0.43–05	N/R	~ 2	No	^38^
Triangular prism	4	~ 5.5 to 20	1.46	1.8	Up to 9	N/R	No	^99^
Triangular prism	4	~ 4.4 to 20	1.56	0.3	Up to 8.9	N/R	No	^99^
Triangular prism	4	~ 3.75 to 20	1.8	0.375	Up to 9	N/R	No	^99^
Core–shell sphere	0.532	5.64	*n*_c_ = 1.6, *n*_s_ = 1.84	~ 0.56	~ 28	~ 2.3	No	^10^
Core–shell sphere	100	5	*n*_c_ = 1.33, *n*_s_ = 1.46	0.37–0.91	53.6–261	N/R	No	^100^
Hemispheric shell	0.4	6.25–12.5	1.5	0.455	~ 150	4.25	No	^101^
Hemispheric shell	1	2.5–5	1.5	0.584	~ 45	4.3	No	^101^
Immersed core–shell hemisphere	0.365	~ 24.6	*n*_c_ = 1.44, *n*_s_ = 1.57	~ 0.8 to 1.15	~ 60 to 200	~ 8–23	No	^102^
Immersed flat-top hemisphere	0.325–0.4	~ 24.6	1.57	~ 0.89 to 1	~ 70 to 125	~ 14 to 31.9	No	^102^
Square steps	0.633	~ 0.6 to 1.26	1.46	0.4–0.51	Up to 6	N/R	No	^103^
Ellipsoid	0.4–0.8	~ 1.63 to 3.25	1.458	0.72–0.78	Up to 28.9	N/R	No	^104^
Dome-shaped fiber tip	1.064	~ 28 to 103	*n*_c_ = 1.44, *n*_cl_ = 1.457	~ 0.94 to 3.95	Up to 4.63	N/R	No	^105^
Fiber-coupled spheroid	0.6328	~ 7	1.43–1.59	N/R	~ 26 to 40	N/R	No	^106^

*DS* Dirac semi-metal, *LC* liquid crystal, *N/R* Not reported, *n*_*c*_ core index, *n*_*s*_ shell index, *n*_*cl*_ cladding index.

The proposed structures made of Cd_3_As_2_ may be associated with certain considerations. For example, due to the high toxicity of Cd and As, extra caution is required in handling of these elements during the synthesis. Although various fabrication methods for Cd_3_As_2_ have been reported, a high-yield cost-effective large-scale fabrication method has not been established yet, unlike the case for semiconductor chip fabrication.

## Conclusions

Through FDTD numerical simulation, we demonstrated formation of tunable 3D photonic jets in terahertz spectral domain (terajets, TJs) by dielectric spheres, cubes, and cylinders coated with a BDS layer. The dependency of the TJ’s main characteristics (FD, FWHM, intensity, and length) on the BDS’s Fermi energy and incident uniform beam’s polarization was explored for each case. Polarization of the illuminating light was found to play an important role on TJ characteristics. Simulation of the TJs under oblique incident demonstrated formation of asymmetric focused beams with the maximum intensity of the TJs at a non-trivial morphology-dependent source-angle.

## Data Availability

The datasets used and/or analyzed during the current study are available from the corresponding author on reasonable request.
